# 3DLRA: An RFID 3D Indoor Localization Method Based on Deep Learning

**DOI:** 10.3390/s20092731

**Published:** 2020-05-11

**Authors:** Shuyan Cheng, Shujun Wang, Wenbai Guan, He Xu, Peng Li

**Affiliations:** 1 School of Computer Science, Nanjing University of Posts and Telecommunications, Nanjing 210023, China; b17121207@njupt.edu.cn (S.C.); b17040101@njupt.edu.cn (S.W.); b17090126@njupt.edu.cn (W.G.); lipeng@njupt.edu.cn (P.L.); 2 Jiangsu High Technology Research Key Laboratory for Wireless Sensor Networks, Nanjing 210003, China

**Keywords:** RFID, three-dimensional localization, deep learning, Internet of Things

## Abstract

As the core supporting technology of the Internet of Things, Radio Frequency Identification (RFID) technology is rapidly popularized in the fields of intelligent transportation, logistics management, industrial automation, and the like, and has great development potential due to its fast and efficient data collection ability. RFID technology is widely used in the field of indoor localization, in which three-dimensional location can obtain more real and specific target location information. Aiming at the existing three-dimensional location scheme based on RFID, this paper proposes a new three-dimensional localization method based on deep learning: combining RFID absolute location with relative location, analyzing the variation characteristics of the received signal strength (RSSI) and Phase, further mining data characteristics by deep learning, and applying the method to the smart library scene. The experimental results show that the method has a higher location accuracy and better system stability.

## 1. Introduction

In recent years, with the development and maturity of satellite navigation systems, it is no longer difficult to achieve high-precision outdoor positioning. However, due to the complex indoor positioning environment, with numerous obstructions, it is difficult to obtain more accurate location information from targets in an indoor environment, which is also a hot research topic.

At present, the common indoor positioning technologies are mainly Wi-Fi, Bluetooth, infrared, ultra-wideband, Radio Frequency Identification (RFID), ZigBee, etc., and mainly divided into two service providers: base station and inertial position [1]. Commercial technologies basically use wireless communication base station solutions, such as Wi-Fi and RFID. RFID technology has the characteristics of a long reading distance, strong penetration ability, anti-pollution, high efficiency, and large amount of information [2]. In this paper, we propose an RFID three-dimensional indoor positioning scheme based on deep learning. Radio Frequency Identification (RFID) technology has the advantages of no contact, permanent storage, strong readability, etc. It can effectively support indoor positioning. Using the unique identification characteristic of the tag to the object, the RFID positioning system obtains the position information of the electronic tag according to the signal sent by the electronic tag received by the reader. While saving costs, it can obtain more authentic and effective target location information. RFID is widely used in various scenes, such as smart libraries, logistics warehouses, shopping malls, etc.

RFID system is mainly composed of hardware components and software components [3]. The hardware components mainly include a reader and RFID tags. The RFID tags are placed on an identified object. The reader generally includes a high-frequency module, a control unit, and a coupling element. Interactive data are inquired between the reader and the RFID tags through radio frequency to realize short-distance communication. The data is sent to a computer through the software components. 

RFID positioning technology is divided into absolute positioning and relative positioning [4]. Absolute positioning refers to the physical absolute positioning of a designated target, while relative positioning refers to determine the positional relationship among the targets. In specific indoor applications, relative positioning and absolute positioning are applied to different scenes. The absolute positioning error is relatively larger, and the approximate position of the object in the coordinate system can be obtained through positioning, while the relative positioning makes it easy to get the positional relationship between different labels, so by attaching the labels to the objects, we can detect and correct the position of the wrong sequence objects.

In real life, RFID indoor localization technology is widely used in warehouse goods inventory, book placement order inspection, etc. We often need to locate a large number of targets, and the data we get for positioning is also very large. For deep learning, the more data used for training, the more accurate its final predictions can be. Therefore, the combination of deep learning and indoor positioning technology can effectively extract high-level and abstract features from the original data, allowing the computer to interpret and predict the data, thus improving the positioning efficiency.

In this paper, a scheme called 3DLRA—combining deep learning with RFID positioning technology—is proposed. The antenna groups are used to obtain the information of the tag to be tested and the reference tag. Through the variation characteristics of RSSI and Phase, the similarity between the tag to be tested and the reference tag is calculated to obtain the absolute position of the tag to be tested on a two-dimensional plane. Then, deep learning is used to mine the characteristics and laws of the data, thus further obtaining the Z-axis position information of the tag with higher accuracy. The relative positioning and absolute positioning are combined to obtain the three-dimensional positioning information of the tag to be tested.

In the following, the research status of the RFID positioning technology is descripted in Section 2; the whole system architecture and model is built in Section 3; the whole experiment is described and analyzed in Section 4; our system is compared with other typical RFID positioning methods in Section 5; and the summary of this paper is given in the last section.

## 2. Related Work

Today’s RFID indoor positioning schemes can be divided into one-dimensional absolute or relative positioning, two-dimensional planar positioning, and three-dimensional spatial positioning. Among them, three-dimensional spatial positioning is a difficult and hot topic in today’s research and has great research value. The following part will elaborate and analyze the existing research results.

The one-dimensional positioning scheme can obtain the absolute position or relative position relation of the target object, and is suitable for the management and recording of goods in assembly line situations. In the indoor environment, because the position of the target object will change, the relative positioning method is usually determined by the position information of the target object. The traditional relative positioning method STPP [5], based on the space–time phase profile, analyzes the spatial order of the tag through the phase curve. The PRDL [6] method proposed by Shen et al. breaks through the bottleneck of high density tags, combines deep learning with relative positioning, and improves the relative positioning accuracy. The HMRL [7] method uses the change in the tag signal caused by human movement to realize high-precision relative positioning.

The two-dimensional positioning scheme can obtain the x–y coordinates of the target, which is suitable for plane navigation technology, etc. The two-dimensional positioning can be divided into ranging positioning and non-ranging positioning. The basic ranging methods include time of arrival (TOA), time difference of arrival (TDOA), angle of arrival (AOA), and received signal strength (RSSI) [8]. For the first time, the LANDMARC [9] system introduces a reference tag, and uses the “K-Neighbor” algorithm to weigh the coordinates of the tags to obtain the position information of the target. The VIRE [10] method improves the LANDMARC method by adding a virtual reference tag and setting a threshold filter tag to simplify the calculation process. BVIRE improved the VIRE algorithm and proposed the concept of a boundary virtual reference tag, which improved the positioning accuracy of the boundary tag to be tested [11]. The ANTspin [12] is introduced into the rotating antenna to dynamically collect the signal strength information of the tag, and the target position information is obtained through calculation. Fu proposed a combination of RFID phase and laser-based clustering to use particle filters to locate the moving object [13]. The LF [14] RFID system uses an LF magnetic field for reliable positioning in multipath and non-line-of-sight environments. 

The three-dimensional positioning scheme can obtain the spatial position information of the target, which is beneficial for the progress of indoor navigation technology and makes people’s life more convenient. The APM [15] scheme handles distance estimation parameters based on minimum interrogation power and multilateral measurements, while the APAA [15] scheme supports reader mobility and promotes a highly dense tag environment. The 3DinSAR [16] method uses a holographic atlas to obtain target position information through the phase difference of tags with different heights. Although this method can realize real-time perception, the calculation is complicated. The VLM three-dimensional localization algorithm [17] is based on virtual tags and topological constraints, but this algorithm has blind spots, which may lead to localization failure. The Tagspin [18] method allows tags to rotate uniformly on the edge of the rotating disk, enabling fast and high-precision positioning. When estimating the received power, multidimensional scaling (MDS) [19] considers path loss and shielding effects, and implements 3D positioning of active RFID tags. The Active–Passive algorithm is based on the Nelder–Mead nonlinear optimization method, which can accurately locate the x-axis and y-axis positions of the tags, but the z-axis positioning accuracy is relatively bad [20].

## 3. System Design

### 3.1. System Architecture

**Figure 1:**
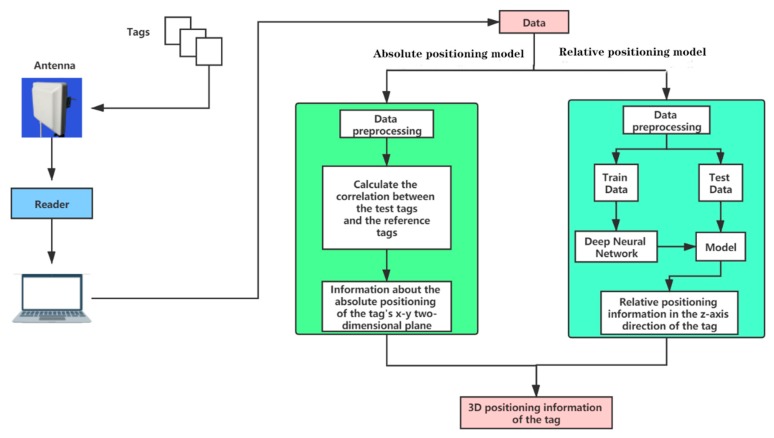
Overall system architecture.

Figure 1 is the overall architecture of the RFID three-dimensional positioning method based on deep learning proposed in this paper. The tag to be positioned and the reference tag send their own information (including antenna port number, EPC number, RSSI, Phase, Timestamp, etc.) to the reader through the antenna. EPC is Electronic Product Code. [6] The server reads the data from the reader and preprocesses the data. Then, the correlation between the reference tags and the tags to be tested is calculated through a mathematical method to obtain the position of the tags to be tested in the two-dimensional plane. In our system, there are some specific location areas in the two-dimensional plane that are meaningful. We need to determine which area each tag to be tested belongs to. We abstract these location areas into points; the location of the tag to be tested is equivalent to the location of the abstract points in the area to which it belongs. In addition, we take the relevant information of the tag to be tested as training data, input the neural network, and output the model; then, we read the test data, input the model, and output the relative position information of the tag to be tested in the Z-axis direction. Combined with absolute positioning and relative positioning, the three-dimensional position information of the tag can be obtained. When calculating the accuracy rate of the three-dimensional positioning, according to the specific application scene of the system, the demand proportion of the absolute positioning and relative positioning of the system is determined, and the accuracy rate of the three-dimensional positioning is obtained by using a weighting algorithm.

### 3.2. Analysis and Forecast

The following information of the radio frequency tag can be obtained through reading by the reader: EPC is a coding system, which is the Code of Electronic products. It builds on the basis of the EAN.UCC (global uniform identification system) barcoding, and the barcoding system has been extended to realize the marking of a single item. The measurement antenna port number, Phase, signal strength (RSSI), Doppler frequency, and Timestamp can be obtained by the RFID system [21]. The following section analyzes the information of the selected RFID tags based on the system requirements. We collected tag data with a certain trend by moving people between fixed antennas and tags. The RSSI, Phase, and timestamp of the tag vary as the person passes by. When people pass through the fixed antenna in a single direction from far to near, the RSSI of the tag increases from large to small and then larger, the Phase changes periodically, and the timestamp gradually increases. In the system, since the tags on the bookshelf are linearly arranged on the first layer of the bookshelf, the variation trend of the RSSI, Phase, and timestamp of the tags is closely related to the order of the tags. We amplify the sequence of the tag data information changes by means of convolutional neural networks to get the order of the tags in the first layer. We consider the moving speed of the human to improve the robustness of the system, so that the system is still accurate under the condition of the different moving speeds of the human.

• **RSSI**

When we need to know the absolute position information of the tag to be measured in space, we can estimate the position of the tag to be measured from the position of the reference tag. The signal strength of the reference tag and the tag to be tested will be read by the reader. When the tag to be measured is close to a reference tag, their signal strength values will be very close to under the same propagation environment and the same transmission loss model [22], so the signal strength of the tag can be used as information for positioning the absolute position of the x–y plane.

**Figure 2:**
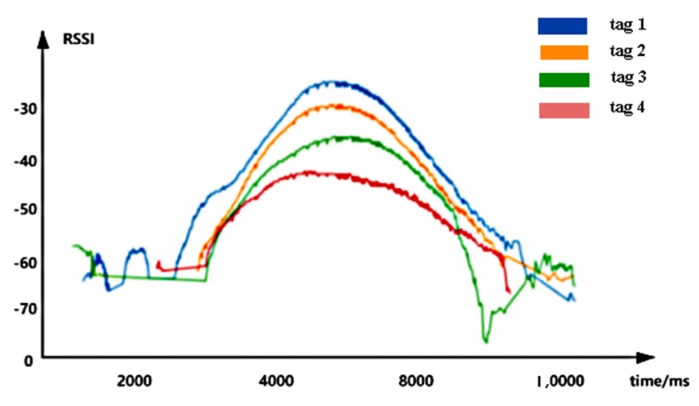
Variation rule of the received signal strength (RSSI) of tags in the same column.

Through a simple experiment, we found that the RSSI has obvious change rules in three-dimensional space: We placed 20 passive tags at equal distances on each layer of the same shelf, for a total of four layers. Set the antenna to face the highest layer, move at a uniform speed, and scan the tags. Finding the RSSI peak value of the same column of the tags occurs at the same time, and the peak value is inversely proportional to the distance from the tag to the antenna. In Figure 2, the X-axis represents the timestamp and the Y-axis represents the RSSI. The curves in Figure 2 shows the change of the RSSI of the tags of the same column from the highest layer to the lowest layer in order from top to bottom. In the figure, the maximum peak value of the blue RSSI curve indicates that the tag 1 is closest to the antenna, while the minimum peak value of the red RSSI curve shows that the tag 4 is the farthest from the antenna and is located at the bottom layer. According to the above RSSI change rule, we think that using RSSI information is very helpful to distinguish the relative relation of tags in height, so we choose the RSSI information of the tags to locate the relative position of the tag to be tested on the shelf. The size of the peak is inversely proportional to the distance from the tag to the antenna. Indeed, if the tags in the same column are only a few centimeters apart, the gap between the RSSI of the tags will be smaller. Therefore, we adopted deep learning to locate the tag on the z-axis according to the slight difference between the tag data; that is, which layer of the shelf the tag is on.

• **Phase**

The Phase is a parameter that defines a position (point in time) in a cycle of a repetitive waveform, a scale that shows whether it is in a crest, trough, or at some point between them. Define the phase measurement *θ* of the reader output as a periodic function, which can be expressed as

(1) $$\theta =\left(2\pi \frac{2L}{\lambda }+\theta_{T}+\theta_{R}+\theta_{Tag}\right)mod\;2\pi$$

where L is the distance between the reader and the tag; λ is the wavelength; and the round-trip distance from the transmission to the reception of each signal is 2L. $\theta_{T},\theta_{R}$, and $\theta_{Tag}$ are the phase shifts introduced by the reader’s transmitting circuit, the reader’s receiving circuit, and the tag’s reflection characteristics, respectively [5]. The space–time phase is used for analysis. The phase distribution of the tags is convenient to obtain the spatial relative order of the tags to be tested. We used a high-frequency RFID with a wavelength of about 22 m; in the library, the distance between the tags on the same shelf was no more than that, so we used Phase as part of the data information to locate the relative position of the z-axis of the tags to be tested.

• **Timestamp**

Timestamp is the time interval between when a reader first reads a tag and when it reads the tag again. To obtain a certain amount of tag data information, it is necessary to increase the reading time of the reader. The change of time is an essential consideration regarding the positioning factors; the objective law of changes of the RSSI and phase information of the tags at different times gives great inspiration for the establishment of the system model, so we chose the Timestamp of the tags as the basic data.

### 3.3. System Model

We set M = 120 passive tags to be tested in three-dimensional space, and set N = 18 as the reference tags near them. We used the Impinj-R420 UHF-RFID reader and five antennas. Four stationary antennas were selected to read the reference tags and related information of the tags to be tested. A person moves between the antennas and the tags to be tested to obtain the information of the tags.

#### 3.3.1. Absolute Positioning Model

• **Preprocess Data**

Take the EPC–RSSI–Timestamp information of the tested tags and reference tags read by the A = 4 antennas as a set of data, read a total of n = 250 sets of data. The data of RSSI and Phase we got are discrete and fluctuate in one dimension. We considered the use of filter methods to deal with it. After the experiment, we finally used wavelet de-noising to reduce data fluctuations and retaining the changing trends of the original. First, we used the MATLAB function to decompose the signal, compute the threshold value. Then, we did global threshold processing, compressing the one-dimensional signal to eliminate the interference of external adverse factors as much as possible [23]. After dealing with the errors in the data, in theory, each of the four antennas can read all the tag information, but considering the actual situation, there may be errors in the information reading process. If some tags are missed by some readers, the RSSI value of the tag is 0. Normally, the RSSI value of the tag read by the reader is negative. It has an impact on the subsequent calculation of the correlation between the reference tags and the tags to be tested. In order to eliminate this effect, we removed the missed tags and reference tags in advance to ensure the integrity of subsequent data.

• **Calculate Data**

We put reference tags around the tags to be tested, read the RSSI strength of each tag with a reader, and got the similarity among the reference tags and the tags to be tested. Based on these similarities, the absolute position of the x-axis of the tag was determined. The system sets *M* tags to be tested, *N* reference tags, and A antennas to read the relevant information of the reference tags and the tags to be tested. Defining the signal strength of the tag to be tested as a matrix *S*, $S_{ij}$(i = 1, 2... M, *j* = 1, 2... *A*) represents the RSSI value of tag *i* to be located and read by the antenna *j*. [24].

(2) $$S=\left[\begin{array}{cccc} S_{11} & S_{12} & \cdots & S_{1A}\\ S_{21} & S_{22} & \cdots & S_{2A}\\ \vdots & \vdots & \vdots & \vdots \\ S_{M1} & S_{M2} & \cdots & S_{MA} \end{array}\right]$$

Defining the signal strength of the reference tag as a matrix *θ* ($\theta_{ij}$, i = 1, 2,... *N*, *j* = 1, 2,... *A*) represents the RSSI value of the reference tag *i* read by the antenna *j* [24].

(3) $$\theta =\left[\begin{array}{cccc} \theta_{11} & \theta_{12} & \cdots & \theta_{1A}\\ \theta_{21} & \theta_{22} & \cdots & \theta_{2A}\\ \vdots & \vdots & \vdots & \vdots \\ \theta_{N1} & \theta_{N2} & \cdots & \theta_{NA} \end{array}\right]$$

Defining the correlation between the reference tags and the tags to be tested as a matrix *E* ($E_{ij}$, *i* = 1,2,... *M*, *j* = 1,2,... *N*) represents the relationship between the *i*-th tag to be tested and the reference tag *j*, which is derived from the mean square error of their signal strength [25]:

(4) $$E_{ij}=\sqrt{\sum_{K=1}^{A}{\left(S_{ik}-\theta_{jk}\right)}^{2}}$$

We divide the plane evenly into multiple quadrilaterals of the same area. The intersection of the diagonals of each small quadrilateral is considered to be the abstracted point of the small quadrilateral. Define the correlation between the tags to be tested and the abstracted points as a matrix $E^{\prime }$, where $E_{ij}^{\prime }$(*i* = 1,2,…120; *j* = 1,2,…; e = 9) represents the correlation between the tag *i* to be tested and the point *j*. Calculate the correlation between the tag to be measured and all reference tags in the plane area represented by the abstract point. Then, the sum of squares of these correlations is calculated. The root of the result of the previous step is $E_{ij}^{\prime }$.

(5) $$E_{ij}^{\prime }=\sqrt{\overset{\theta }{\underset{K=1}{\sum }}e_{iK}^{2}}$$

The smaller *E*’ is, the higher the correlation between the tag under test and this location area, and the more likely the tag under test is in this location area. Therefore, as long as the minimum value in each row of the matrix is found, it can be determined at which point the position of the tag to be tested, corresponding to this row, is equivalent.

#### 3.3.2. Relative Positioning Model

• **Preprocess Data**

The person moves between the selected antenna and the tags to be tested, and uses the EPC–RSSI–Phase–Timestamp of the tag to be tested as a set of data. A total of 250 sets of data are read, then the data is wavelet de-noised and returned. Normalization processing, specifying the phase range (0,2π), and standardizing the phase information of each tag:

(6) $$P_{s}=\frac{P}{2\pi }$$

The RSSI indicates the strength of the signal received by the antenna. The theoretical maximum value of the RSSI is 0 and its range is (−90, 0). The RSSI is processed as follows:

(7) $$RSSI_{s}=0.01RSSI+1$$

For Timestamp, we set its normalization method as

(8) $$t_{s}=\frac{t-t_{min}}{t_{max}-t_{min}}$$

• **Mining data**

**Figure 3:**
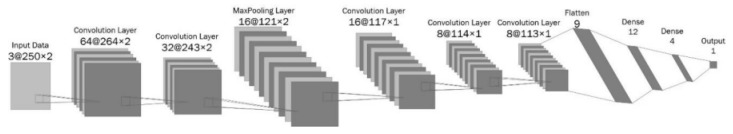
Neural network model.

Convolutional Neural Network (CNN) is a kind of feed-forward neural network. Its artificial neurons can respond to a part of the surrounding cells in the coverage area, and it has excellent performance for large image processing [26]. CNN consists of many convolutions and pooling, followed by an optional fully connected layer. CNN can simulate feature differentiation through convolution and through the weight sharing and pooling of the convolution; reduce the order of magnitude of the network parameters; and complete the classification through traditional neural networks [27]. Compared with other neural networks, such as LSTM, we find that CNN can effectively meet the needs of our system, so we finally chose a CNN-based neural network. The neural network model is shown in Figure 3. There are five convolutional layers, one pooling layer, two fully connected layers, and the output layer activation function. For Softmax, there is a Dropout layer in the neural network, and the final output is the absolute tomographic information of each tag.

**Figure 4:**
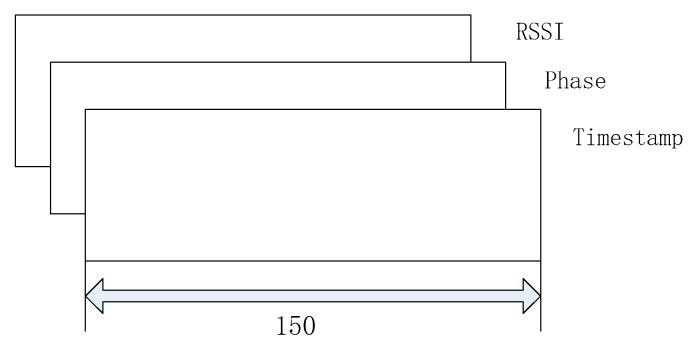
Convolutional layer input data.

In the experiment, we used the RSSI, Phase, and Timestamp of the tag as the input data to extract more abundant data features of tag when a person passed the antenna at a constant speed. The input of the convolution layer is data of A×B×C, where A×B is the data dimension of label in experiment, and C is the number of channels; that is, the dimension of the rag’s data characteristics, and the data of the tags have three channels, which are the characteristics RSSI, Phase, and Timestamp, as shown in Figure 4. 

The convolutional layer has k convolution kernels of size m×m. These convolution kernels determine the area size of each neuron in the convolutional layer and the adjacent neurons in the previous layer [28]. When the neural network works, it regularly scans the characteristics of the input data. In the area where the characteristics of the input data coincide with the convolution kernel, each pixel value is multiplied by the weight of the corresponding point in the convolution kernel. The offset can get a pixel value in the output data [29]. 

**Figure 5:**
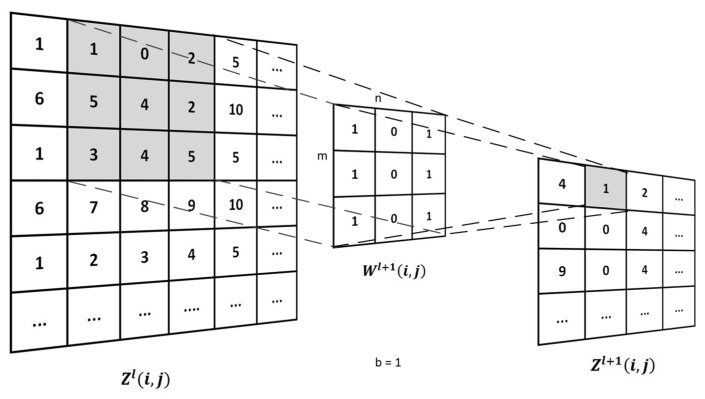
Convolutional operation.

The convolution is when a convolution kernel traverses the input data. The input data is multiplied by the corresponding value in the area where the convolution kernel coincides, and then the sum is added to bias, and finally we get a value of the output data. Figure 5 explains the process of convolution operation. Among them, $Z^{l+1}\left(i,j\right)$ represents the data of the convolution operation layer l+1 neural network, and $Z^{l}\left(i,j\right)$ represents the l layer neural network; *W* is the weight; K is the number of convolution kernels; m is the height of the convolution kernel; and n is the width of the convolution kernel.

(9) $$Z^{l+1}\left(i,j\right)=\left[Z^{l}\left(i,j\right)W^{l+1}\right]\left(i,j\right)+b=\sum_{K=1}^{K}\sum_{x=1}^{m}\sum_{y=1}^{n}Z_{k}^{l}\left(S_{0}i+x,S_{0}j+y\right)$$

The Function Softmax normalizes the gradient logarithm of the discrete probability distribution of finite terms. The input of the function is the result obtained from *K* different linear functions, and the probability P of the sample vector x belonging to the classification j is [30]

(10) $$P\left(y=j|x\right)=\frac{e^{x^{T}w_{j}}}{\sum_{k=1}^{K}e^{x^{T}w_{k}}}$$

In the system, for the relative positioning of the tag to be tested on the z-axis, the input data of the neural network were (*n*, 3, 1, *Q*), where *Q* is the length of the input data, and the data were extracted from the convolution layer for feature extraction. Maximal pooling performs feature selection and information filtering on the output feature maps, and then combines the extracted features non-linearly to obtain the output, using the existing high-order features to complete the learning goal. Through the convolutional neural network, we obtained the information confidence of the z-axis direction of the labelled tag; the maximum value of the confidence of the position information of the tag is taken as the position of the tag in the Z-axis direction.

## 4. System Implementation

### 4.1. Hardware and Software

We mainly used an Impinj-R420 UHF-RFID reader, equipped with five E9208PCRNF UHF antennas and a set of H47 passive tags. The reader was connected to the same Ethernet with the computer through a network cable, and the reader got the data through Tagsee. The computer we used was configured with an Intel Core i7-9750H, memory 8G, and graphics card GTX 1650 4G. The program is programmed with Python 3.6. The Python packages required for the program to run were tensorflow cpu1.1.0, keras2.1.2, numpy1.2.10, pandas0.2.0, and matplotlib1.2.0.

### 4.2. Environmental Deployment

There are many types of books in the library, and manual sorting takes a lot of time and effort. Therefore, RFID positioning technology is commonly used to manage books. In summary, we set the library as an environmental scene, and obtain the bookshelves that you want to find by 3D positioning. With the layer position of the books on the bookshelf, this RFID three-dimensional positioning helps book managers to find books, thereby saving manpower. This method is also applicable to other scenarios, such as warehouse logistics. Considering that the radio frequency in the UHF band is relatively sensitive to the environment, especially metal, we used wooden bookshelves in our experiments.

According to the LANDMARC system, we know that the density of the reference tags will affect the performance of the x–y plane positioning. We have placed a different number of reference tags on each layer of the bookshelf and conducted multiple sets of experiments, as shown in Table 1. Finally, we found that placing three reference tags in each layer is the most reasonable, which can not only save costs, but also obtain a higher positioning accuracy. 

**Figure 6:**
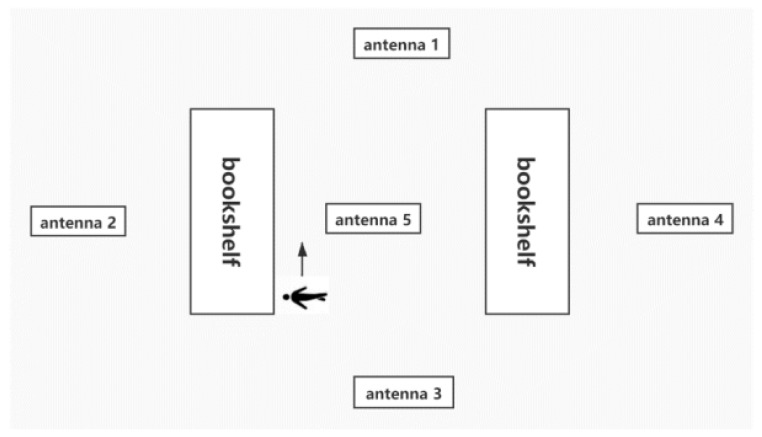
Experimental environment deployment.

**Figure 7:**
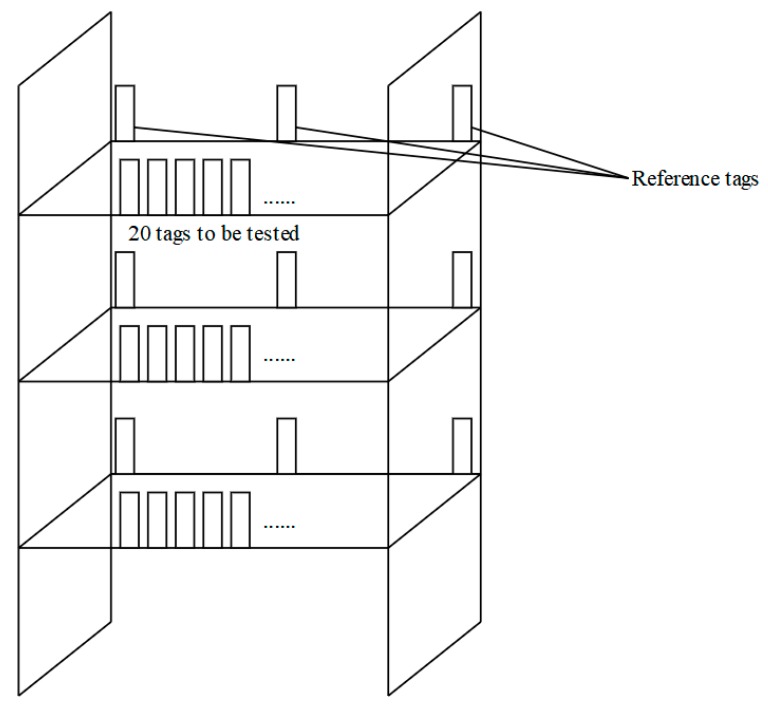
Bookshelf deployment.

Figure 6 shows the experimental scenario we set up, in which five antennas and two bookshelves were placed. Each bookcase is shown in Figure 7. Three layers of passive tags are placed on the bookshelf, and 20 tags are tested on each layer. The distance between the tags to be tested was 2 cm. There were 18 reference tags in total. Three reference tags were placed on each layer of each shelf. They were arranged on both sides and in the middle. On each layer of the shelf, the reference label was placed on the inner side, and the label to be tested was placed on the outer side. The antenna height was 95 cm, and the shelf height was 25 cm. The five antennas were divided into two groups: Antennas 1–4 were Group A, and Antenna 5 was Group B; Group A antennas were used to obtain the absolute position information of the x–y plane of the tag to be tested, and the Group B antenna was used to obtain the z-axis relative position information of the tag to be tested.

### 4.3. Data Collection

Group A used four antennas to read the information of the reference tags and the tags to be tested at the same time. Each time it reads for 20 s, and each tag can read up to 250 sets of data by one of the antennas, and saved to realize the positioning of the absolute position of the tag to be tested in the x-y plane. Group B uses one antenna to read the information of the tags. The antenna remains stationary, and one person moves between the bookshelf and Antenna 5. In the original experiment, the distance between the antenna and the tags on the bookshelf was 38.5 cm; the person moved 180 cm, and the person passed along the tag sequence at a uniform speed. The movement time was about 12 s. Each time, an average of 60 tags were collected, and each tag had about 100 sets of data. In order to effectively process the data, the data of the tags were complemented with 150 pieces by using the method of zero padding, as data backup.

### 4.4. Data Processing

We imported the collected data of Group A into the absolute positioning model of the system. In the smart library, because we need to know which shelf a book is on, we put a reference tag on each shelf. Through the RSSI read by each reader, the similarity between the tag on the book and all reference tags was obtained, and we computed the minimum correlation to integrate these similarities. The specific method is mentioned in Section 3.3.1. Then, the similarity between the tag and each shelf is obtained. We determined which shelf the tag is on by comparing the similarities.

We imported the collected data of Group B into the relative positioning model of the system. The input data of the neural network was (n, 3, 1,150), and the length of the input data was 150. After getting the neural network model, we brought the test data into the model and get the confidence of the layer position information of the tags. The theoretical position information of the tags of the third layer was (0,0,1); the actual location differs from the information confidence. The maximum value of the confidence sequence of the tag was taken as the predicted position information of the actual layer of the tag. 

### 4.5. Analysis of 3D Positioning Accuracy

We defined the absolute accuracy and relative accuracy. Absolute accuracy indicates the proportion of books with the correct absolute position on the x–y plane to the total number of books. The relative accuracy indicates the proportion of books with correct relative positions in the z-axis direction to the total number of books. Considering that the requirements for absolute accuracy and relative accuracy of the tags are different in different scenarios, we used a weighted algorithm to express the final 3D positioning accuracy. We stipulated that R_A represents relative accuracy and A_A represents absolute accuracy. Suppose the proportion of relative accuracy is a, and the proportion of absolute accuracy is b, and a + b = 1. Then, the three-dimensional positioning accuracy is expressed as follows:

(11) Acc=a×R_A+b×A_A

In the smart library scenario, by comparing different segmentation weights, multiple experiments were performed, and each segmentation weight was subjected to 50 experiments, and the experimental accuracy was analyzed, as shown in Table 2. Under the condition that the absolute positioning and the relative positioning are balanced, it was finally found that the weighted operation was performed at a ratio of 8:2, and the obtained 3D positioning accuracy is the highest and the most objective and true. The highest 3D positioning accuracy obtained was 96.264%. In the following calculation, we replaced “b” with 0.2 and replaced “a” with 0.8 in Equation (11). The three-dimensional positioning accuracy is expressed as follows:

(12) Acc=0.8×R_A+0.2×A_A

**Figure 8:**
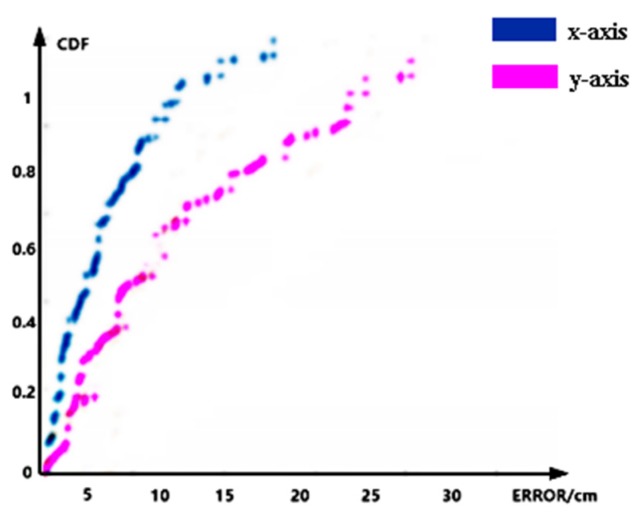
CDF of the errors of the horizontal position.

In addition, we verified the performance of the system in a horizontal position. The deployment of the experimental scenario remains unchanged. We moved the tags to be tested to collect about 150 pieces of test data. Figure 8 is the CDF graph of the absolute positioning error. In the figure, the blue curve indicates that the average positioning error on the x-axis is 3.82 cm, and the pink curve indicates that the average positioning error on the y-axis is 8.35 cm. Combining the average positioning error of the x-axis and y-axis, the average positioning error of the two-dimensional plane is 10.02 cm. The error was better than many existing solutions; e.g., AOA (70 cm) [8], Landmarc (30 cm) [9], and Vire (14 cm) [10].

### 4.6. Robustness Analysis

When implementing the system, we determined the distance between the antenna and the tag sequence, the speed of human movement, and the height of the antenna. In actual applications, these parameters are not determined. This requires us to explore the robustness of the final model and try to improve the stability of the model. We changed the distance between the antenna and the tag sequence, the moving speed of the person, and the height of the antenna; then, we collected multiple sets of data for testing, and observed how the changes in these parameters affect the positioning accuracy. 

• **Distance between antenna and tags**

In the experiment, the distance between Antenna 5 and the tags was 38.5 cm. On this basis, the distance was increased or decreased to analyze the change of the relative positioning accuracy. The results are shown in Table 3. We first conducted experiments in the range of increasing or decreasing 1–5 cm. Considering the power of the antenna, we set the maximum distance between the antenna and the tags to 53.5 cm, and the closest distance to 23.5 cm. Within the set variation range, the overall accuracy was greater, above 0.9. Therefore, the system is more robust in terms of the distance between the antenna and the tags.

• **Speed of moving**

In the experiment, we set the speed of human movement between eleven and twelve centimeters per second, and adjusted it based on this. Since the speed of human movement cannot be accurately controlled, we increased the number of experiments to ensure the objectivity of the result. As shown in Table 4, the results show that the overall accuracy is high and the model is robust.

• **Antenna height**

In the experiment, we set the height of the antenna to 95 cm, and adjusted it based on this. It is considered that if the change of the antenna height is small, it has almost no effect on the accuracy, so the antenna height change range, as shown in Table 5, was set. After experimental verification, we can see that the system is robust.

## 5. System Comparison

Based on the experimental scenes, we used the methods mentioned in the following table to test the positioning accuracy. Finally, we found that PRDL has a higher accuracy in one-dimensional positioning, combining with deep learning. Using similar values, HMRL has a higher accuracy in two-dimensional positioning. LANDMARC has high accuracy in the two-dimensional positioning by using the reference label. ANTspin uses a rotating antenna combined with deep learning to achieve a high accuracy three-dimensional positioning. Active–Passive locates objects in 3D space by using RFID tags and readers. VLM provides fine-grained localization accuracy in 3D positioning based on connectivity information. 3DLRA combines the characteristics mentioned above achieving a higher accuracy in three-dimensional positioning.

Below is a comparison of our 3D positioning method with existing classic RFID positioning solutions. “√” in Table 6 indicates that the positioning method has the characteristics of this column. 

From the analysis of the above table, we can see that the RFID 3D positioning scheme we designed has certain advantages over previous studies. Our positioning system does not require moving antennas, which can save manpower and costs. In addition, we used deep learning technology in combination with absolute positioning and relative positioning to further mine data features, thereby obtaining a higher positioning accuracy. 

## 6. Summary

This paper proposes a kind of RFID three-dimensional positioning scheme. The system combines relative positioning and absolute positioning, uses deep learning technology, and uses the RSSI, Phase, and Timestamp of the tags to train the model, finally achieving better positioning results. Through experimental verification, the stability of the positioning model is good, and it can be applied to real-life scenarios. The development prospect of the system can be widely used in many indoor positioning environments.

## Figures and Tables

**Table 1:** Influence of the density of the reference tags on the accuracy of plane positioning.

Number of Reference Tags on Each Layer	Positioning Accuracy
1	57.132%
2	75.374%
3	86.543%
4	87.432%

**Table 2:** Accuracy of the 3D positioning under different segmentation proportions.

Segmentation Proportions	Positioning Accuracy
5:5	92.415%
6:4	93.698%
7:3	94.981%
8:2	96.264%

**Table 3:** Distance between the antenna and tags.

Distance between Antenna and Tags	Positioning Accuracy
23.5 cm	0.938
28.5 cm	0.953
33.5 cm	0.975
35.5 cm	0.981
36.5 cm	0.989
37.5 cm	0.991
39.5 cm	0.992
40.5 cm	0.987
41.5 cm	0.973
43.5 cm	0.963
48.5 cm	0.954
53.5 cm	0.932

**Table 4:** Speeds of movement.

Speed of Movement (cm/s)	Positioning Accuracy
9–10	0.938
11–12	0.952
13–14	0.941

**Table 5:** Antenna height.

Antenna Height	Positioning Accuracy
80 cm	0.928
85 cm	0.953
90 cm	0.975
92 cm	0.981
93 cm	0.989
94 cm	0.991
96 cm	0.992
97 cm	0.987
98 cm	0.973
100 cm	0.963
105 cm	0.952
110 cm	0.931

**Table 6:** Comparisons of RFID positioning methods.

RFID Positioning Method	3D Positioning	No Need to Move the Antenna	Combining Deep Learning	High Accuracy of Positioning
PRDL [6]			√	√
HMRL [7]		√		√
LANDMARC [9]				√
ANTspin [12]	√		√	√
Active-Passive [20]	√			√
VLM [17]	√	√		√
3DLRA	√	√	√	√
